# Institutional Needs

**Published:** 1913-09-20

**Authors:** 


### Institutional Needs.

### THE PURCHASE OF RUBBER GOODS.

Independent of the wet weather that is always associated with an English autumn and winter, even if the summer happens to be fine, the question of waterproofs, in the form of sheets, "wet dressings," rubber air-goods, and so forth, is a perennial problem to hospital managers. Thus the latest edition of Anderson's Catalogue deserves close attention. The firm's reputation for quality and reasonable price is now as beyond dispute as that of any ordinary enterprise is in the nature to be, so we will confine our attention to certain details of the catalogue which are particularly interesting just now. Operating aprons, gloves, sleeves, and tubing, mats, nursing aprons, waterproof sheeting, elastic-webbing bandages, rubber matting, and *post-mortem* gloves are very clearly catalogued with illustrations and prices, that make a search for the article required and the drawing up of an estimate exceedingly simple. It is also noteworthy that the catalogue is not the size of an encyclopædia several pounds in weight, but capable of being put in comfort on a desk or in an overcoat pocket. The firm's address is, of course, 37 Queen Victoria Street, E.C.

### PROPAESIN.

(B. Kuhn and Co., 16 Rood Lane, London, E.C.)

A new local anæsthetic, to which the name of Propaesin has been given, has been placed upon the market by the Hamburg firm of Frans Fritsche and Co., and can be obtained in London from B. Kuhn and Co. at the above address. Chemically it is the propyl ester of para-amidobenzoic acid; it is a white crystalline powder, very slightly soluble in water, but easily soluble in alcohol and ether. It has a slightly bitter taste, and almost at once produces anæsthesia of the lips and tongue. Used as a powder for insufflation into the larynx in cases of tuberculous laryngitis, it has met with marked success; and Dr. Karl Perl, of Berlin, has given it internally in doses of 7½ grains for gastric ulcer, corrosive poisoning, and similar conditions. Drs. Stürmer and Lüders, of Hamburg, were the investigators whose work resulted in this compound, and they set to work on a definite plan. Like Ehrlich with salvarsan, they took into consideration the best grouping of radicles calculated to yield a satisfactory local anæsthetic, and then synthesised seven separate esters of para-amidobenzoic acid. Somewhat contrary to the expectations based on theoretical grounds, the propyl and amyl esters turned out to have the most powerful anæsthetic actions: the former was, at the same time, non-irritating and non-toxic, whereas the amyl compound did not possess those advantages. It would seem that the chief field of usefulness for Propaesin will probably lie in the alleviation of pain in stomatitis, pharyngitis, tuberculosis, syphilis, malignant disease, ulcer, and so on, rather than in procuring local anæsthesia for minor operative surgery.

### BILTON'S PATENT MILK AND BUTTER COOLER.

Science is nothing if not simple, and in an age which is weary, especially in the institutional section of its life, of elaborate and expensive mechanical devices for doing the simplest thing, Bilton and Co.'s Patent Milk and Butter Cooler is something of a revelation. It is even attractive to the eye. A red-brown outer bowl, like a round flower-pot in appearance, loosely encloses a white-glazed clay vessel with a closely fitting cover. The space between the two is filled with water which forms a jacket. In the inner vessel milk or butter is placed, and the cooling, as our experience can testify, is efficiently effected by the oozing of the water through the porous outer vessel, where it evaporates, and in so doing absorbs the heat from the butter within. It is, of course, advisable to stand the whole cooler in a soup-plate or shallow dish, as, after a night's process of evaporation, the place on which the cooler is stood becomes wet. This exceedingly simple and attractive contrivance has one limitation only, that, namely, it can be used for small quantities of milk or butter alone. On the other hand this is its intention, for in a large institution or household heavy refrigerators are, of course, needed to do all the work required. In the ward or private room, on the other hand, a cheap, efficient, small, portable cooler is precisely what is wanted. Bilton and Co.'s patent costs only a few shillings, and is made in several sizes, and can be sincerely recommended for the type of need already explained. It may be added that in cold weather the outer vessel can be used as a jardinière, and the inner, since it possesses a cover, will always be a useful adjunct to the ward, kitchen, or store room. The address of the makers is Fenton, Stoke-on-Trent.

